# Microstructured Hollow Fiber Membranes: Potential Fiber Shapes for Extracorporeal Membrane Oxygenators

**DOI:** 10.3390/membranes11050374

**Published:** 2021-05-20

**Authors:** Paul Ecker, Markus Pekovits, Tsvetan Yorov, Bahram Haddadi, Benjamin Lukitsch, Martin Elenkov, Christoph Janeczek, Christian Jordan, Margit Gfoehler, Michael Harasek

**Affiliations:** 1 Institute of Chemical, Environmental and Bioscience Engineering, TU Wien, 1060 Vienna, Austria; markus.pekovits@tuwien.ac.at (M.P.); bahram.haddadi.sisakht@tuwien.ac.at (B.H.); benjamin.lukitsch@tuwien.ac.at (B.L.); christian.jordan@tuwien.ac.at (C.J.); michael.harasek@tuwien.ac.at (M.H.); 2 Institute of Engineering Design and Product Development, TU Wien, 1060 Vienna, Austria; tsvetan.yorov@tuwien.ac.at (T.Y.); martin.elenkov@tuwien.ac.at (M.E.); christoph.janeczek@tuwien.ac.at (C.J.); margit.gfoehler@tuwien.ac.at (M.G.)

**Keywords:** sherwood number, computational fluid dynamics, extracorporeal membrane oxygenators, micro- particle image velocimetry

## Abstract

Extracorporeal membrane oxygenators are essential medical devices for the treatment of patients with respiratory failure. A promising approach to improve oxygenator performance is the use of microstructured hollow fiber membranes that increase the available gas exchange surface area. However, by altering the traditional circular fiber shape, the risk of low flow, stagnating zones that obstruct mass transfer and encourage thrombus formation, may increase. Finding an optimal fiber shape is therefore a significant task. In this study, experimentally validated computational fluid dynamics simulations were used to investigate transverse flow within fiber packings of circular and microstructured fiber geometries. A numerical model was applied to calculate the local Sherwood number on the membrane surface, allowing for qualitative comparison of gas exchange capacities in low-velocity areas caused by the microstructured geometries. These adverse flow structures lead to a tradeoff between increased surface area and mass transfer. Based on our simulations, we suggest an optimal fiber shape for further investigations that increases potential mass transfer by up to 48% in comparison to the traditional, circular hollow fiber shape.

## 1. Introduction

The use of respiratory assistance devices for patients with severe forms of respiratory failure, such as extracorporeal membrane oxygenators, allow for low tidal volume protective ventilation, therefore reducing the stress associated with mechanical ventilation [1]. Improving the efficiency of hollow fiber membrane oxygenators is a crucial topic as the survival rate for patients is low (between 60 and 70% [2]), which is partially contributed to by the large amount of blood that is circulated out of the body and into the membrane module [3]. Therefore, a potential way to optimize oxygenator performance is to increase the membrane area available for CO_2_ and O_2_ gas exchange, without increasing the priming volume of the device. One way to achieve this improved area-to-volume goal is the use of microstructured hollow fiber membranes that alter the traditional circular shape of the membrane surface.

**Figure 1:**
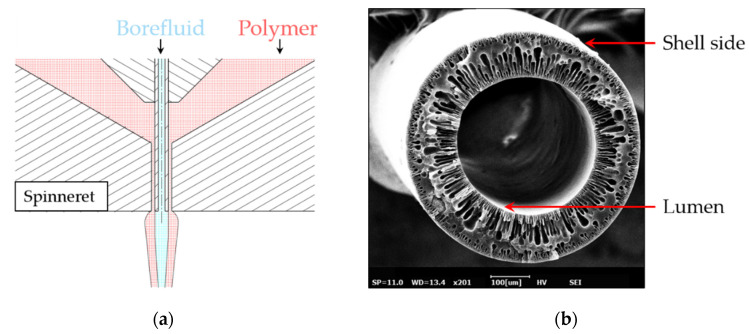
Hollow fiber membrane terminology: (**a**) Schematic of a spinneret for membrane production. (**b**) Scanning electron microscopy cross section of a hollow fiber membrane with circular lumen and shell side.

Hollow fiber membranes are commonly produced by utilizing a phase inversion process, where a liquid polymer solution is pumped through a ring gap with a non-solvent solution (“Borefluid”) in the center (Figure 1a) [4]. Adjustment of the spinneret allows for a microstructured lumen or shell side of a fiber (Figure 1b). A number of studies altered the lumen geometry of hollow fibers either by directly adjusting the spinneret [5] or spinning parameters [6]. However, for applications in membrane oxygenators, microstructuring of the lumen is less important, as the main transport resistance occurs on the blood and, therefore, shell side of the fiber [1].

Experimental work that altered the shell side of the fiber in the longitudinal direction, using a pulsating bore fluid concept, showed potential improvements in the mass transfer capabilities in comparison to a straight fiber geometry [7,8]. In a different approach, microstructuring was achieved by rotating a 3D printed spinneret, resulting in helically twisted fiber geometries [9]. Both the pulsating and rotating concepts induce microstructuring along the fiber, while keeping the traditional circular fiber cross section of the spinneret. Another method to enhance the fiber surface would be to adapt the cross section of the spinneret, which increases the options for non-cylindrical fiber shapes. Experimentally, this has been done by Çulfaz et al., who investigated the influence of spinning parameters on the shape of a structured ultrafiltration fiber [10].

Augmenting the fiber shape not only increases surface area, but also changes blood flow characteristics around the hollow fibers. As with any membrane separation process, secondary flow structures should be encouraged, and stagnating zones, where the convective mass transfer is inhibited, should be avoided to reduce the risk of concentration polarization [11]. This is especially true for blood-contacting applications where areas of low flow velocity are a potential source for thrombus formation. A thrombus is an agglomeration of red blood cells and platelets that, if big enough and detached from the vessel walls, can cause critical complications such as cerebral infarction or pulmonary embolism [12]. Detailed knowledge about the flow field around microstructured fibers is therefore valuable for the selection of an optimal fiber shape, however, little work has been published in this regard. Yang et al., used computational fluid dynamics (CFD) to evaluate different fiber shapes for direct contact membrane distillation. They predicted a gear-shaped cross section to achieve the highest average mass flux, however, they limited their research to a straight single-fiber module [13].

Therefore, the question arises: Is there an optimal fiber shape that maximizes membrane surface area and increases mass transfer, while simultaneously not increasing the risk of potential flow stagnation zones? As the production of arbitrary shaped hollow fiber membranes is complex, and experimental visualization of the flow patterns inside a hollow fiber membrane packing is difficult [14], computational fluid dynamics simulations are a potentially powerful tool to gain insight to this question. In this work, we follow the approach of Santos et al. [15] to calculate the local Sherwood number on a membrane surface as a qualitative measure of mass transfer. In total, we examine seven different geometries, theoretically increasing the available gas exchange surface by up to 79% compared to the traditional, circular shape. For this study, we chose an experimental design that represents transverse flow through an oxygenator hollow fiber packing. Initially, we compared experimental velocity data from micro- particle image velocimetry (µPIV) measurements to computational fluid dynamics results in order to validate our simulations. By examining the computed flow field and Sherwood numbers, we give a discussion on potentially adverse flow structures and calculate theoretical oxygenator performance.

## 2. Materials and Methods

### 2.1. Non-Circular Fiber Shapes

In total, seven different cases were evaluated: A circular fiber geometry in a non-staggered arrangement (“Circle, non-staggered”), a circular fiber geometry in a staggered arrangement (“Circle, staggered”) and five non-circular geometries in staggered arrangements (Table 1).

Non-circular geometries were created using a sinusoidal function (Equation (1)), that incorporated the average diameter (*d_avg_*), which was kept constant at 400 µm, amplitude (*x*), number of periods (*n*) and angle (*ϕ*).

(1) $$d\left(x,n,\phi \right)=d_{Avg}+x\times sin\left(n\times \phi \right)$$

**Figure 2:**
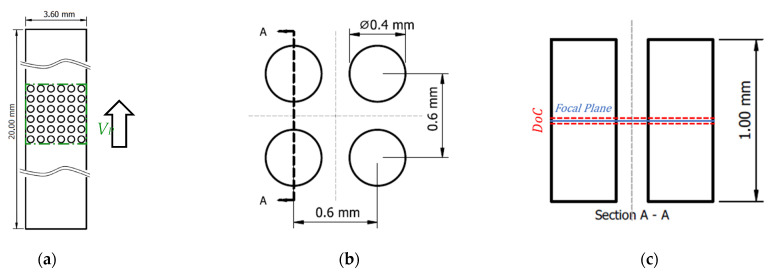
Dimensions of the micro- particle image velocimetry (µPIV) experiment: (**a**) Overall dimensions of the channel and number of fibers, arrow denotes flow direction. Green line outlines packing volume *V_P_*; (**b**) fiber dimensions and non-staggered arrangement; (**c**) side view of fiber arrangement and location of focal plane. Depth of correlation (DoC) not to scale.

The specific area *S* (Equation (2)) was calculated as the total membrane surface area *A* in relation to the packing volume *V_P_* (Figure 2a, green line).

(2) $$S=\frac{A}{V_{P}}$$

### 2.2. Experimental Setup

In order to approximate transverse flow conditions in a membrane oxygenator, a rectangular channel (3.6 mm × 20 mm × 1 mm) with a non-staggered fiber arrangement in the center was fabricated (Figure 2a). Diameter (400 µm) and center to center distance (600 µm) between the fibers correspond to typical dimensions found in hollow fiber membrane oxygenators [16] (Figure 2b). A 6 × 6 arrangement placed in the center was chosen to eliminate possible influence from the channel walls and ensure fully developed flow profiles. Fabrication of the acrylic channel and fiber arrangement was done using CNC milling. Using digital microscopy (VHX-6000, Keyence, Osaka, Japan), the quality of manufacturing in regard to dimensions was verified. Finally, the channel was sealed by gluing a thin acrylic sheet on the top that provided optical access to the flow chamber for µPIV measurements.

For this study, deionized water was used as a working fluid (μ = 1 mPas). A syringe pump (Harvard Apparatus Model 11, Instech Laboratories Inc., Plymouth Meeting, PA, USA) controlled the flow rate during the experiment. Selection of the flow rates was based on previous work [16] that allowed estimation of fluid velocity between fibers in a prototype hollow fiber membrane module (0.42 mL/min, 0.72 mL/min and 1.29 mL/min). Using the average inlet velocity *U*, fiber diameter *d* and kinematic viscosity *ν*, the Reynolds number (Equation (3)) for the performed experiments corresponded to 0.8, 1.3 and 2.4.

(3) $$Re=\frac{Ud}{\nu }$$

### 2.3. Velocity Measurement

**Figure 3:**
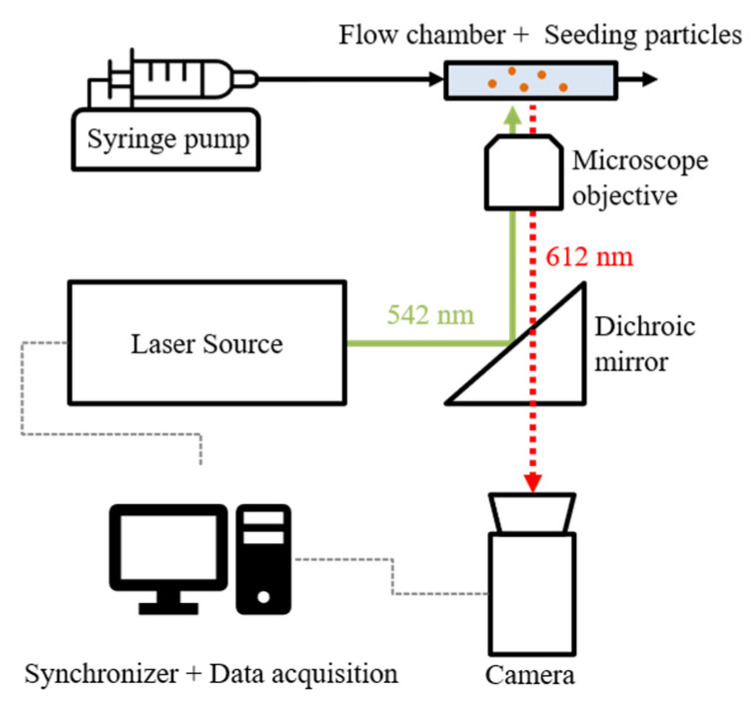
Schematic of the µPIV measurement principle.

A micro- particle image velocimetry (µPIV) system was used to visualize the flow velocity field between two fibers in the arrangement. A simplified schematic of the measurement principle is given in Figure 3. In µPIV, a fluid flow is seeded with fluorescent tracer particles that follow the flow field. Two quick, successive laser pulses are used to excite fluorescent signals that are observed by a camera. Knowing the timing between pulses, imaging processing software calculates the velocity field based on the movement of the particles. Detailed explanation of this measurement principle is given elsewhere, for example, in [17].

The system used here consisted of a Nd:YAG laser (Bernoulli 200-15, Litron Lasers Ltd., Rugby, Warwickshire, UK) with emission at 532 nm in combination with an inverted microscope (Olympus IX73, Tokyo, Japan) and a high-speed camera (Zyla 5.5 sCMOS USB 3.0, Andor, Oxford Instruments plc, Tubney Woods, Abingdon, UK). The camera control input was connected to a synchronizer (LaserPulse Synchronizer 610036, TSI Inc., Shoreview, MN, USA), which adjusted the camera shots to the laser pulses. The output of the camera was connected to the control PC unit where the imaging software processed the results (4G Insight 11.1.0.5, TSI Inc., Shoreview, MN, USA). The flow channel was fixed on the stage of the microscope. Approximately 5 v% polystyrene seeding particles with a diameter of 1.8 µm were added to the working fluid. The excitation peak of the fluorescent dye was 542 nm and the emission peak was 612 nm (Fluoro-Max, Thermo Fisher Scientific, Fremont, CA, USA). Postprocessing and image generation of the results were performed using Tecplot 360 (Tecplot Inc., Bellevue, WA, USA). The depth of correlation (DoC), i.e., the distance above and beneath the focal plane where particles were illuminated [17], was calculated as 30 µm.

**Figure 4:**
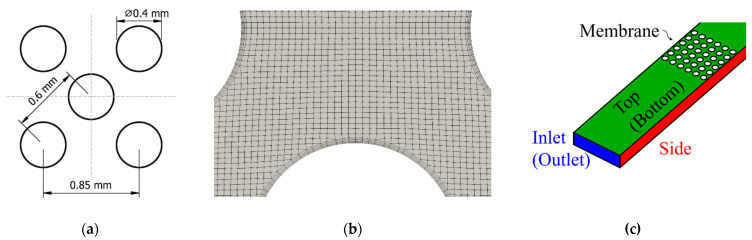
(**a**) Dimensions of staggered arrangement; (**b**) detail of the mesh for circular and staggered geometry showing the boundary layers along the membrane wall; (**c**) schematic of the boundary assignment, names in brackets denote opposite boundary faces.

Velocity measurements were performed at the center plane (height 500 µm) of the channel, between two fibers (Figure 2c). For the validation of the CFD simulations, the velocity magnitude was extracted along the centerline between two fibers (Figure 4a, white dotted line). Following this approach, repeated measurements were performed, and the measurement error was calculated.

### 2.4. Computational Fluid Dynamics

Computational domains were derived from the experimental setup (Figure 2a), which changed only in the shape of the fibers according to Table 1. All non-circular fiber shapes were arranged in a staggered pattern where the distances between the fiber centers were kept constant (Figure 4a). Spatial discretization, or meshing, was done using the mesh generation utility snappyHexMesh [18]. A mesh dependence study evaluating the influence of cell size on the mean Sherwood number was performed, resulting in about 500,000 cells for all geometries (see Appendix B). Special care was taken with the membrane patches to ensure uniform boundary layers along the surface (Figure 4b), as the calculation of the Sherwood number is reliant on the gradient in this area.

Based on the inlet Reynolds number (Equation (3), hydraulic diameter as characteristic length), laminar flow was expected throughout the computational domain, therefore no turbulence model was selected. The computational domain consisted of patches for inlet, outlet, membrane and wall structures (Figure 4c). Inlet velocity boundary conditions were derived from experimental flow rates by calculating the average velocity. The open source code OpenFOAM^®^ 5.0 (The OpenFOAM Foundation Ltd., London, England) [17] was used for the computational fluid dynamics simulations. All simulations were run on server nodes equipped with 32 core CPUs (16 cores in two physical modules, EPYC 7351, AMD). Postprocessing and extraction of velocity data were completed by ParaView 5.8.0 (Kitware, Inc., Clifton Park, NY, USA) [19]. 

#### 2.4.1. Flow Simulations

A steady state, incompressible solver (simpleFoam) using the semi-implicit method for the pressure linked equations algorithm (SIMPLE) with second order discretization schemes was applied to solve the governing equations for momentum and mass conservation (Equations (4) and (5)), that characterize the flow field for an incompressible, Newtonian fluid. These simulations were carried out until the convergence criteria for pressure and velocity were met (residuals < 1 × 10^−5^).

(4) ∇×(U)=0

(5) (U×∇)U−νΔU=−∇p/ρ

Two types of flow simulations were conducted. First, simulations where the velocity field was compared to the experimental µPIV data (“Validation simulation”). Second, simulations to generate the velocity field for the Sherwood number calculations (“Sherwood simulations”). For the validation simulations, a no-slip boundary condition for velocity and zero gradient boundary condition for pressure were applied on all wall structures. This was done to match the flow conditions within the experimental microfluidic channel. In contrast, Sherwood number simulations applied cyclic boundary conditions at the top and bottom wall, i.e., were treated as neighboring patches [20]. The reasoning behind this approach was to simulate mass transfer on a continuous fiber packing, eliminating non-physical wall effects for the calculation of the Sherwood number. Boundary conditions for both simulation types are summarized in Table 2.

#### 2.4.2. Sherwood Number Simulations

After convergence for pressure and velocity was achieved, the resulting velocity field was mapped to the computational domain and a second, transient solver comprising Equations (6)–(8) (modified version of scalarTransportFoam) was used to calculate the local Sherwood number on the membrane patches. This was done by solving the transport equation for an arbitrary component *T* (Equation (6)), where *D_T_* denotes the diffusion coefficient of *T*. In this work, *D_T_* is set as 6.96 × 10^−10^ m^2^/s, which corresponds to the diffusion of dissolved CO_2_ in blood [16].

(6) $$\frac{\partial T}{\partial t}+\nabla \left(UT\right)-\nabla^{2}\left(D_{T}T\right)=0$$

The local mass transfer coefficient *k_c_* of each cell was then calculated by the surface normal gradient of *T* (Equation (7)).

(7) $$k_{c}=-\frac{D_{T}}{T_{b}}\frac{\partial T}{\partial y}{|}_{y=0}$$

Finally, the local Sherwood number of each membrane face was calculated (Equation (8)), where *d* is the average fiber diameter of the structure.

(8) $$Sh=\frac{k_{c}d}{D_{T}}$$

A maximum Courant number limit of 1 was chosen to adjust time steps in these simulations [21]. Termination was done after no significant change in the Sherwood number was observed (~3000 time steps). A fixed inlet concentration of 1, and - as an approximation - complete removal on the membrane walls were assumed for species *T* (Table 3). 

### 2.5. Evaluation of Results

**Figure 5:**
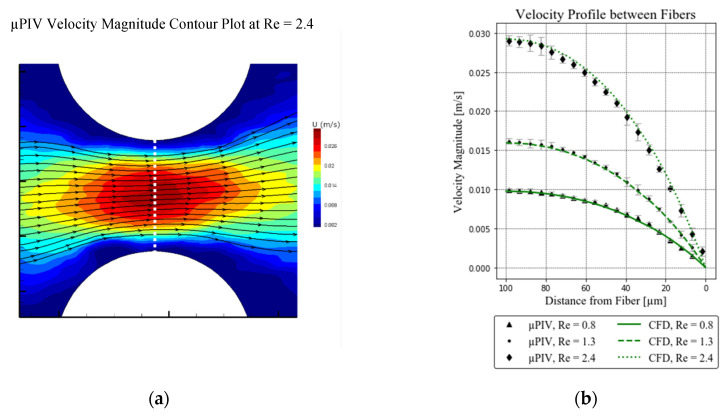
Experimental results: (**a**) Contour plot of the processed µPIV measurement at Re = 2.4; (**b**) comparison of CFD and µPIV velocity magnitude along the center line, only half of the symmetric velocity profile is shown.

Experimental (µPIV) and numerical (CFD) velocity magnitudes were compared by extracting flow profiles along the center plane of the channel. Positioning on the x-axis was done by matching maximum velocities of the parabolic flow profiles (Figure 5b). Subsequently, the percentage mean error was calculated as a measure of fit between experimental and numerical data. 

To assess the CFD results, the area-weighted averaged Sherwood number was calculated by the total membrane area of the computational domain *A*, the local Sherwood number of a cell *Sh_i_* and face area of that cell *a_i_* (Equation (9)).

(9) $$\bar{Sh}=\frac{\sum_{i}^{n}Sh_{i}\;a_{i}}{A}$$

As a means to compare the flow conditions of the different geometries, the velocity distribution was computed for the CFD data (Equation (10)). This was done by relating the volume fraction of cells that included velocities of a certain category ($\sum_{i}^{n}v_{i}$) to the total volume fraction of the fiber packing ($V_{P})$. Only cells inside the fiber packing were considered for this calculation.

(10) $$U_{Frac}=\frac{\sum_{i}^{n}v_{i}}{V_{P}}$$

In order to evaluate the influence of the fibers varying in specific area, we calculated the theoretical flux of component T *(J_T_*) for different oxygenator module sizes ranging from 100–300 mL, which approximately corresponded to priming volumes found in adult membrane oxygenators [22]. Calculation was done as shown in (Equation (11)), where *A* is the membrane surface derived from the specific area, and ΔT the driving force of component *T*, i.e., difference between surface and bulk value. As an approximation, we set the concentration of *T* on the membrane walls to zero, assuming total removal of the component. The mean mass transfer coefficient $\bar{k_{C}}$ was determined based on the CFD results.

(11) $$J_{T}=A\times \bar{k_{C}}\times \Delta T$$

## 3. Results

### 3.1. µPIV Measurements

The visualized, experimental flow field between two fibers in the center of the packing at Re = 2.4 is given in Figure 5a. Velocity magnitude is presented as a contour plot with streamlines depicting flow direction. In Figure 5a, low velocity areas close to the fiber walls are clearly visible. Extraction of the velocity magnitude along the white dotted line yields the experimental flow profile depicted in Figure 5b. Error bars denote measurement uncertainty derived from three repeated measurements. Numerical results are presented as green line plots. The mean and maximum deviations between experimental and numerical data for the individual flowrates are as follows: Re 0.8: mean 2.6%, max. 8.3%; Re 1.3: mean 1.9%, max. 12.7%; Re 2.4: mean 6.1%, max. 11.2%.

### 3.2. Computational Fluid Dynamics Results

**Figure 6:**
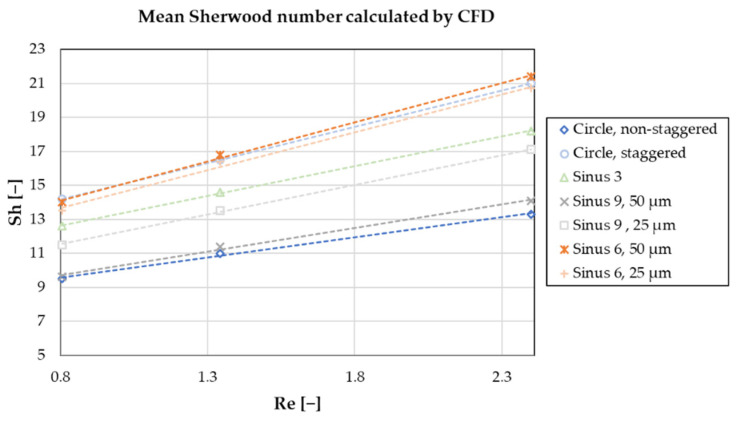
Mean calculated Sherwood number for all investigated structures and arrangements.

The mean Sherwood number as calculated by (Equation (9)) in relation to the Reynolds number is given in Figure 6. All geometries show a clear linear increase in the mean Sherwood number with increasing Re (R^2^ > 0.98), however, the slope of this function varies. At lower Re, the differences between the geometries are less pronounced than at high Re. Ranking the geometries, we observe the best results, i.e., highest Sherwood number, in Sinus 6 50 µm, circular staggered and Sinus 6 25 µm options. The lowest values are observed in the circular non-staggered and Sinus 9 50 µm arrangements.

**Figure 7:**
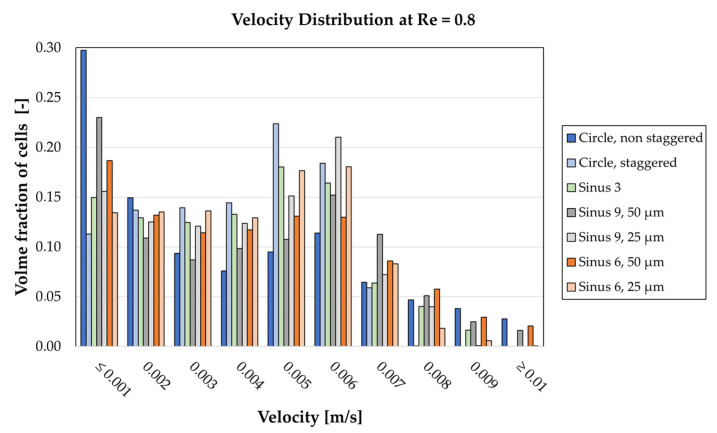
Comparison of velocity distribution from 0.001 to 0.01 m/s at Re = 0.8. Only cells inside the packing of the computational domain were considered.

**Figure A1:**
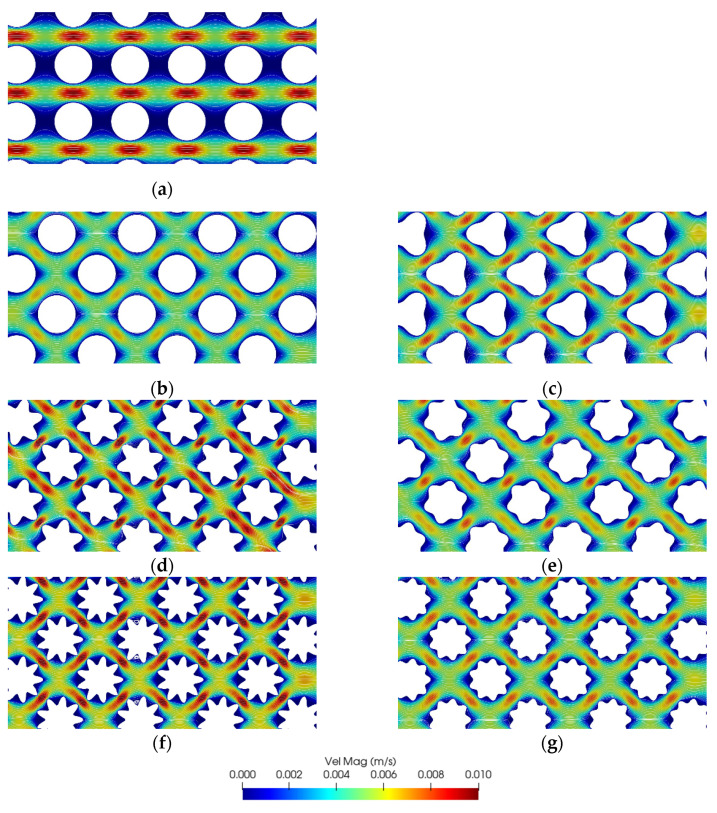
CFD velocity results for Re = 0.8. Flow direction from left to right. (**a**) Circle, non-staggered; (**b**) Circle, staggered; (**c**) Sinus 3; (**d**) Sinus 6, 50 µm; (**e**) Sinus 6, 25 µm; (**f**) Sinus 9, 50 µm; (**g**) Sinus 9, 25 µm.

The velocity distribution inside the different fiber packings for a Reynolds number of 0.8 is given in Figure 7, according to Equation (10). The highest volume fraction at velocities below 0.001 m/s is found in the circular, non-staggered arrangement at almost 30%, whereas staggering these fibers results in the lowest amount in this category at about 12%. On the other side of the spectrum, we find that only three of the seven geometries include velocities that exceed 0.01 m/s (circular non-staggered, Sinus 6, 50 µm and Sinus 9, 50 µm). Overall, the circular staggered arrangement yields the most uniform velocity distribution. Excluding the lowest velocity category, we find the modal value of all geometries between 0.005 and 0.006 m/s for this Reynolds number. As a visual comparison of the flow fields, CFD velocity contour plots of all geometries are given in Appendix A (Figure A1) for Re 0.8.

**Figure 8:**
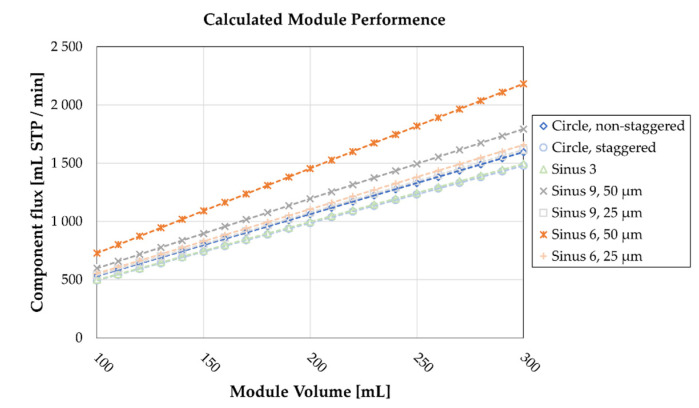
Calculated module performance at Re = 0.8 for varying membrane module volume sizes, assuming total component removal on the membrane surface. Mass transfer coefficient calculated by CFD.

Employing Equation (11), we calculate a theoretical module performance for different oxygenator volumes at Re 0.8 (Figure 8). With increasing module size, the differences in performance are increased. We observe the lowest performance in the staggered circlular and Sinus 3 options. The best performance, standing out from all other geometries, is the Sinus 6, 50 µm variant. Comparing best and worst performing geometries, a difference of about 50% in component flux is observed.

## 4. Discussion

The aim of this study was the detailed investigation of the flow field around microstructured hollow fiber membranes and calculation of their theoretical mass transfer capabilities. Initially, we conducted µPIV experiments on one of the structures to validate the velocity field obtained by our CFD simulation. Comparison of the velocity magnitude given in Figure 5b shows good agreement between experimental and numerical data, with a maximum deviation between CFD and µPIV of 12.7%. To account for uncertainty caused by the depth of correlation, CFD data were extracted not only at the center plane, but also at positions corresponding to the DoC (focal plane ± 30 µm, as indicated in Figure 2c). Notably, however, due to the height of the channel (1 mm), this variation caused only minor changes in the results and was therefore deemed negligible for this investigation. Both the experimental (Figure 5a) and numerical (Figure A1a) velocity contour plots show high velocities between the fibers in the flow direction, and low velocity regions perpendicular to the flow. This influences the velocity gradient along the membrane surface, which in turn influences the Sherwood number.

Looking at Figure 6, we find that the slope (*k*) of the Sherwood number in relation to the Reynolds number varies between the geometries. It is lowest in the Circle non-staggered (*k* = 2.4), and highest in the Sinus 6, 50 µm (*k* = 4.6) variant. Higher Reynolds numbers, equal to higher blood flow rates through the oxygenator packing, are therefore beneficial to increase mass transfer and potentially impact the effectiveness of microstructured fibers. Additionally, we found that the Sherwood number does not increase with an increasing number of periods (Sinus 6 > Sinus 3 > Sinus 9). In regard to amplitude, there is a clear difference between the Sinus 6 and Sinus 9 geometry. For Sinus 6, both the 25 and 50 µm variants result in similar Sherwood numbers. Contrary, for Sinus 9, a difference of about 20% is observed between the 25 and 50 µm options. These findings indicate interactions between the number of periods and amplitude, suggesting an ideal combination for maximum Sherwood number.

The velocity distribution inside the fiber packings is of great interest for the present investigation for two main reasons. First, concentration polarization, the buildup of a concentration gradient in the membrane boundary layer, reduces membrane efficiency and should therefore be avoided. One way to prevent this phenomenon is the disruption of the boundary layer by induction of secondary flows, while low-velocity, stagnating zones should be avoided [11]. Whereas concentration polarization can be assumed as a general challenge in membrane separation processes, hemostasis and subsequent thrombus formation are unique to applications in blood-contacting devices. The formation mechanism of thrombi is complex, however, a major contributing factor is areas of low blood flow [23]. Therefore, we use the velocity distribution given in Figure 7 as a measure of thrombosis risk, i.e., the higher volume in the lowest velocity category (≤0.001 m/s), the higher the risk for hemostasis. Judging by this criterion, the least risk for thrombosis would be found in the Circle, staggered and the highest risk in the Circle, non-staggered geometry. Notably, the amplitude plays an important role in this regard as both the Sinus 6 and Sinus 9 geometries contain more low-velocity volume when their respective amplitude size is 50 µm as compared to 25 µm. Furthermore, we observe a correlation between the number of periods and low-velocity areas as structures with three, six and nine 50 µm amplitudes show corresponding increases in low-velocity volume (fraction ≤ 0.001 m/s: Sinus 3: 15%, Sinus 6, 50 µm: 19%, Sinus 9, 50 µm: 23%).

**Figure 9:**
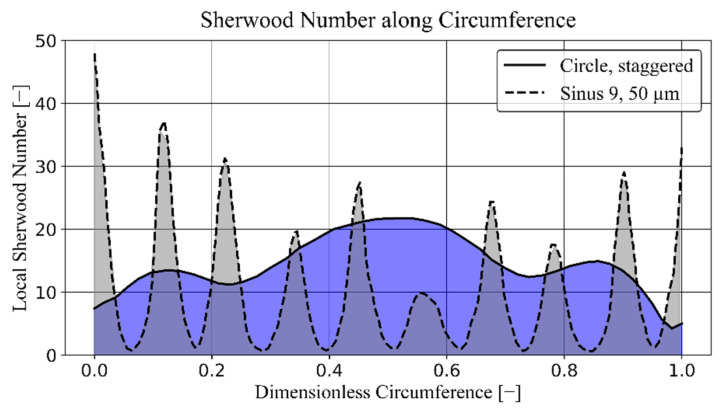
Local Sherwood number along the circumference of two different single fibers.

Looking at the CFD contour plots of the velocity flow fields (Figure A1), low-velocity zones are found around the fibers and inside the amplitudes. Using the local Sherwood number calculated on the membrane surface, we can visualize this observation by plotting along the circumference of a single fiber (Figure 9). For example, geometries with low (Circle, staggered) and high (Sinus 9, 50 µm) fractions of low-velocity zones are compared. Clearly, the high velocities between the nine amplitudes create periodic, pointwise high Sherwood numbers. However, these alternate with areas of stagnating flow, causing the Sherwood number to drop significantly. On these parts of the membrane surface, convective mass transport would be close to zero.

The influence of these low Sherwood number regions is apparent when comparing expected and actual calculated increases in component flux (Table 4). Using the Circle, staggered geometry as a baseline, the microstructured fiber shapes increase the available surface area at a constant volume by up to 79%. If no changes in mass transfer coefficient were assumed, these increases would reflect the expected performance increase. Comparing these values to the calculated component fluxes (Figure 8), where the mass transfer coefficient is derived from the CFD data, differences are obvious. Primarily, across all structures, the actual increase is lower than the expected one, which contributes to the low Sherwood number regions around the fibers. Notably, we find the lowest differences in the geometries with six periods, and the highest in the geometries with nine periods, i.e., there is no corresponding increase in gas exchange performance with an increasing number of periods. In general, we found that an increase in specific area does not lead to an equivalent increase in component flux.

Out of the possibilities investigated in this work, we propose that the Sinus 6, 50 µm geometry is the most suitable potential shape for a microstructured hollow fiber. With a calculated increase in component flux of 48%, it surpasses the other possibilities by a wide margin. Moreover, the velocity distribution of this variant shows moderate fractions of low-velocity regions, which reduces additional risks of thrombosis. Therefore, it is the most promising candidate for future spinning of a microstructured hollow fiber membrane.

### Limitations of This Study

The findings of this study are of potential interest for future membrane oxygenator optimizations, however, limitations apply. First, the geometry in this work approximates real-world membrane packings accounting for transverse flow but neglecting parallel flow along the fibers. In this regard, we follow previous investigations in this field [24,25]. Furthermore, this arrangement was chosen as it allows the use of µPIV measurements to visualize the experimental flow field and subsequently validate our CFD results. Due to the nature of the measurement principle, flow parallel to the fiber axis is difficult to measure.

Second, the Sherwood number-based model in this work is a simplified approach to compare mass transfer in hollow fiber membranes that assumes total removal of the species on the membrane walls. It does not account for permeances, solubility or partial pressure of the components. Including these factors in the modeling of membrane mass transfer is an important research topic addressed by numerous publications [26,27], however, this is not the aim of this work. The present approach allows for a qualitative, but not quantitative, comparison of different fiber structures.

As whole blood cannot be used for µPIV measurements due to its optical properties, we used water as the working fluid for the present investigation. Although essentially a non-Newtonian fluid, the shear thinning properties of blood are only present at low shear rates (<200 s^−1^) [28]. These shear rates are usually exceeded in membrane packings [29], allowing it to be treated as a Newtonian fluid. We checked this assumption in our simulation, comparing Newtonian and Casson viscosity models [30], and found no difference in results.

Lastly, the results of this work are solely based on the shell side geometry of hollow fiber membranes, neglecting the potential influence of the lumen shape. It is obvious that a combination of a circular lumen with any of the alternative shapes presented here would lead to very inconsistent wall thicknesses, which in turn would lead to varying mass transfer along the fiber circumference. Consequently, we note that the application of microstructured fibers probably requires the same geometric shape for the shell and lumen side of hollow fiber membranes. Assuming a phase inversion process for the production of fibers, this implies equal adjustment of both the bore and dope fluid part of the spinneret.

## 5. Conclusions

Improving mass transfer in oxygenators by introducing microstructured hollow fibers with a larger surface area is a plausible way to increase performance. In an effort to find a fiber shape that maximizes mass transfer but at the same time reduces the risk of flow-stagnating zones, we conducted validated computational fluid dynamics simulations to calculate the local Sherwood number on the membrane surfaces and evaluate flow conditions around the fibers. We found that amplifying the area-to-volume ratio bears the risk of creating low-flow areas around the fibers which, apart from potential concentration polarization, increases risk for thrombus formation. Based on the simulation results, we conclude that increasing the specific area by adjusting membrane shell surfaces does not automatically lead to increased oxygenator performance. From the structures investigated in this work, the Sinus 6, 50 µm option showed the most promising result, increasing the calculated component flux by up to 48% compared to the circular geometry.

## Figures and Tables

**Table 1:** Overview of the five non-circular fiber shapes investigated in this work. All non-circular shapes are organized in a staggered arrangement.

Name	Unit	Sinus 3	Sinus 6, 50 µm	Sinus 6, 25 µm	Sinus 9, 50 µm	Sinus 9, 25 µm
Cross section	-	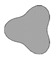	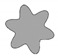	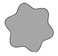	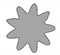	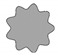
Average Diameter	µm	400	400	400	400	400
No. of Periods	-	3	6	6	9	9
Amplitude	µm	50	50	25	50	25
Specific Area	m^2^/m^3^	3810	4976	3920	5962	4482

**Table 2:** Boundary conditions applied in the computational fluid dynamics (CFD) flow simulations.

Boundary	Validation Simulation		Sherwood Simulation	
	Velocity	Pressure	Velocity	Pressure
Inlet	uniform value	zero gradient	uniform value	zero gradient
Outlet	zero gradient	uniform value	zero gradient	uniform value
Membrane	no-slip	zero gradient	no-slip	zero gradient
Side wall	no-slip	zero gradient	no-slip	zero gradient
Top and bottom wall	no-slip	zero gradient	cyclic	cyclic

**Table 3:** Boundary conditions applied in the CFD simulations for Sherwood number calculations.

Boundary	Specie, *T*
Inlet	uniform value, 1
Outlet	zero gradient
Membrane	uniform value, 0
Side wall	zero gradient
Top and bottom wall	cyclic

**Table 4:** Comparison of the expected and actual component flux increase (Equation (11)). Percentile values refer to a comparison with the “Circular, staggered” geometry.

Geometry	Expected Increase Based on Area ^1^	Actual Increase Based on Sherwood ^1,2^	Difference
	[%]	[%]	[%]
Sinus 3	15	1	−14
Sinus 9, 50 µm	79	21	−58
Sinus 9, 25 µm	35	9	−26
Sinus 6, 50 µm	50	48	−2
Sinus 6, 25 µm	18	12	−6

^1^ In relation to the Circle, staggered geometry. ^2^ Differences calculated at Re = 0.8 and 300 mL module volume.

**Figure A2:**
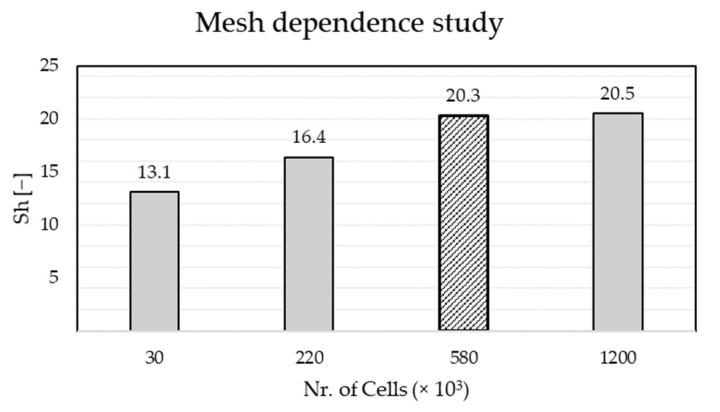
Influence of mesh cell size on the mean Sherwood number for the circular, staggered geometry at Re = 2.4. Highlighted mesh size was chosen for this investigation. Number of cells rounded to two digits.

## Nomenclature

**Acronyms**	
CFD	Computational fluid dynamics
µPIV	Micro- particle image velocimetry
DoC	Depth of correlation
Re	Reynolds number [−]
*Sh*	Local Sherwood number [−]
$\bar{Sh}$	Mean Sherwood number [−]
**Latin Symbols**	
*A*	Membrane surface area [m^2^]
*J_T_*	Component flux [mL/min]
*S*	Specific area [m^2^/m^3^]
*T*	Arbitrary species [m^3^/m^3^]
*T_b_*	Bulk value of species T [−]
*U*	Velocity [m/s]
*k_c_*	Local mass transfer coefficient [m/s]
$\bar{k_{c}}$	Mean mass transfer coefficient [m/s]
*D_T_*	Diffusion coefficient [m^2^/s]
*U_Frac_*	Velocity magnitude fraction [−]
*v_i_*	Cell volume [m^3^]
*V_P_*	Packing volume [m^3^]
*d*	Fiber diameter [m]
*a_i_*	Cell face area [m^2^]
*x*	Amplitude [m]
*n*	Number of periods [−]
**Greek Symbols**	
*ρ*	Density [kg/m^3^]
*μ*	Dynamic viscosity [mPas]
*ν*	Kinematic viscosity [m^2^/s]
*ϕ*	Angle [rad]
